# Adjuvant Therapy for High-Risk Stage II or III Colon Adenocarcinoma: A Propensity Score-Matched, Nationwide, Population-Based Cohort Study

**DOI:** 10.3390/cancers11122003

**Published:** 2019-12-12

**Authors:** Chia-Lun Chang, Kevin Sheng-Po Yuan, Alexander T.H. Wu, Szu-Yuan Wu

**Affiliations:** 1 Department of Hemato-Oncology, Wan Fang Hospital, Taipei Medical University, Taipei 106, Taiwan; richardch9@hotmail.com; 2 Department of Otorhinolaryngology, Wan Fang Hospital, Taipei Medical University, Taipei 106, Taiwan; dryuank@gmail.com; 3 Ph.D. Program for Translational Medicine, Taipei Medical University, Taipei 110, Taiwan; chaw1211@tmu.edu.tw; 4 Department of Food Nutrition and Health Biotechnology, College of Medical and Health Science, Asia University, Taichung 413, Taiwan; 5 Division of Radiation Oncology, Lo-Hsu Medical Foundation, Lotung Poh-Ai Hospital, Yilan 265, Taiwan; 6 Big Data Center, Lo-Hsu Medical Foundation, Lotung Poh-Ai Hospital, Yilan 265, Taiwan; 7 Department of Healthcare Administration, College of Medical and Health Science, Asia University, Taichung 413, Taiwan; 8 Department of Radiology, School of Medicine, College of Medicine, Taipei Medical University, Taipei 110, Taiwan

**Keywords:** colon adenocarcinoma, mortality, fluoropyrimidine, FOLFOX, FOLFIRI

## Abstract

*Purpose:* To determine the optimal adjuvant chemotherapy regimen for patients with high-risk stage II or III colon adenocarcinoma, we conducted this propensity score-matched, nationwide, population-based cohort study to estimate the effects of adjuvant treatments in high-risk stage II or III colon adenocarcinoma. *Patients and Methods:* Using propensity score matching, we minimized the confounding effects of sex, age, pathologic stage, tumor location, total chemotherapy cycles, and Charlson comorbidity index scores on adjuvant treatment outcomes in patients with high-risk stage II or III resectable colon adenocarcinoma. We selected the patients from the Taiwan Cancer Registry database and divided them into four groups: Group 1, comprising patients who received surgery alone; group 2, comprising those who received adjuvant fluoropyrimidine alone; group 3, comprising those who received adjuvant oxaliplatin-fluoropyrimidine-leucovorin (FOLFOX); and group 4, comprising those who received adjuvant folinic acid-fluorouracil-irinotecan (FOLFIRI). *Results:* In both univariate and multivariate Cox regression analyses, the adjusted hazard ratios (aHRs, as well as the 95% confidence intervals (Cis)) for mortality observed for groups 1, 2, and 4 relative to group 3 were 1.55 (1.32 to 1.82), 1.22 (1.05 to 1.43), and 2.97 (2.43 to 3.63), respectively. After a stratified subgroup analysis for high-risk stage II colon adenocarcinoma, we noted that the aHR (95% CI) for mortality for group 2 relative to group 3 was 0.52 (0.30 to 0.89). *Conclusions:* Adjuvant fluoropyrimidine alone is the most optimal regimen for patients with high-risk stage II colon adenocarcinoma compared with the other adjuvant chemotherapy regimens. Adjuvant FOLFOX can serve as an optimal regimen for patients with pathologic stage III colon adenocarcinoma, regardless of age, sex, or tumor location.

## 1. Introduction

Colon cancer is a malignant and prevalent disease worldwide [1]. Both environmental and genetic factors influence the risk of colon cancer [2]. In Taiwan, adenocarcinoma is the most common pathologic type of colon cancer, accounting for >90% of cases of colon cancer; the median age at diagnosis of colon adenocarcinoma is 60 years [3], and colon cancer is the most common cancer and the third leading cause of death in Taiwan [3]. In addition, most colon adenocarcinoma tumors are resectable, with the resectable rate being >90% [3]; 18.83% and 22.26% of patients with colon adenocarcinoma are at stages II and III, respectively [3].

Adjutant chemotherapy is strongly recommended after surgery for high-risk stage II or III colon adenocarcinoma in Taiwan [3]. Taiwanese physicians typically follow treatment procedures or recommendations outlined in previous trials, such as the Multicenter International Study of Oxaliplatin/Fluoropyrimidine/Leucovorin in the Adjuvant Treatment of Colon Cancer (MOSAIC), National Surgical Adjuvant Breast and Bowel Project (NSABP) C-07 Trials, or National Comprehensive Cancer Network (NCCN) guidelines [4,5,6,7]. However, adjuvant chemotherapy regimens have not been demonstrated to have favorable survival effects in large samples of Asian patients with high-risk stage II or III colon cancer [5,6,7]. Moreover, the MOSAIC and NSABP C7 trials have not considered treatment with surgery alone or in combination with adjuvant folinic acid-fluorouracil-irinotecan (FOLFIRI) therapy. The MOSAIC and NSABP trials have compared the survival effects of adjuvant oxaliplatin-fluoropyrimidine-leucovorin (FOLFOX) therapy with those of adjuvant fluoropyrimidine alone in patients with high-risk stage II or III colon cancer [5,6]. Randomized controlled trials have not reported clear survival benefits of adjuvant therapy in patients with stage II colon cancer, although the survival benefits of adjuvant therapy in patients with resected stage III colon cancer have been established [5,6,8]. Additionally, no strong evidence exists regarding the survival benefits of adjuvant FOLFOX in elderly patients with stage II or III colon cancer who may have age-related organ function decline and comorbid conditions that may limit life expectancy. For clinicians treating such patients, special attention must be paid to the risks of chemotherapy, including both treatment-related toxicity and quality of life issues [9,10,11,12]. Studies have also debated whether different adjuvant chemotherapy regimens exert different effects on left- and right-side colon cancers because of differences in gene mutation [13,14].

The aim of the current propensity score-matched cohort study was to determine the optimal adjuvant chemotherapy regimen for Asian patients with high-risk stage II or III colon adenocarcinoma regardless of age, pathologic stage, or tumor location.

## 2. Patients and Methods

### 2.1. Data Source

The Taiwan Cancer Registry database (TCRD) established by the Collaboration Center of Health Information Application contains detailed cancer-related information [15,16,17,18,19,20,21,22,23,24]. This database thus constituted the source of data for the present study. Our protocols were reviewed and approved by the Institutional Review Board of Taipei Medical University (TMU-JIRB No. 201402018).

### 2.2. Study Cohort

We established a cohort of patients identified from the TCRD. We enrolled patients who received a diagnosis of colon adenocarcinoma and underwent surgery between 1 January 2006 and 31 December 2014. The index date was the date of surgery. The follow-up duration was from the index date to 31 December 2016. Our protocols were reviewed and approved by the Institutional Review Board of Taipei Medical University. The diagnoses of the enrolled patients were confirmed according to their pathological data, and patients who received a new diagnosis of colon adenocarcinoma and underwent surgery were confirmed to have no other cancers or distant metastasis. We included patients if they were diagnosed as having colon adenocarcinoma with an indication of surgery, were aged ≥20 years, were of Asian ethnicity, and had pathologic cancer stage IIB–IIIC without metastasis according to the 7th edition of the American Joint Committee on Cancer (AJCC) staging manual. We excluded patients if they had a history of cancer before colon adenocarcinoma diagnosis, unknown pathologic types, neuroendocrine neoplasms, hamartomas, mesenchymal tumors, lymphomas, signet ring cancers, mucinous carcinomas, missing sex data, unclear staging, unclear margin status, or nonadenocarcinoma histology. Pathologic stages IIB–IIC in our enrolled patients with high-risk stage II colon cancer include pT4a (involving tumor invasion through the visceral peritoneum) and pT4b (involving direct tumor invasion or adherence to adjacent organs or structures as well as vascular, lymphatic, or perineural invasion) [5,25]. We also excluded patients with colon adenocarcinoma who had less than 12 lymph nodes examined; previously received chemotherapy, immunotherapy, or radiotherapy; received insufficient cycles (≤12) of adjuvant chemotherapy after surgery; or started adjuvant treatment 8 weeks after surgery. All adjuvant treatments started when no recurrence was recorded in the TCRD by Taiwan Cancer Registration professionals. Finally, we enrolled patients with colon adenocarcinoma who received surgery and then categorized them into the following groups according to adjuvant therapy: Group 1, comprising patients who received surgery alone (no adjuvant chemotherapy); group 2, comprising patients who received adjuvant fluoropyrimidine alone; group 3, comprising patients who received adjuvant FOLFOX; and group 4, comprising patients who received adjuvant FOLFIRI. Cetuximab, panitumumab, bevacizumab, and FOLFOXIRI (fluorouracil plus leucovorin, oxaliplatin, and irinotecan) are disallowed in current adjuvant regimens.

### 2.3. Exposure Assessment

Comorbidities were scored using the Charlson comorbidity index (CCI) [26,27]. Only comorbidities observed 6 months before the index date were included. Comorbid conditions were identified and included according to the main International Classification of Diseases, Ninth Revision, Clinical Modification (ICD-9-CM) diagnosis codes for the first admission or 3 or more repeated main diagnosis codes for outpatient department visits.

We applied propensity score matching (PSM) to reduce the effects of confounders. We estimated propensity scores (PSs) by using a multivariable logistic regression model, with the treatment and confounders serving as the dependent variables and covariates, respectively. The confounders were sex, age, pathologic stage, tumor location, total chemotherapy cycles, and CCI score. Through PSM executed using the global optimum method [28], we matched patients in group 4 with those in the remaining groups at a 1:4 ratio.

### 2.4. Endpoint

The endpoint was the mortality rate of patients who received adjuvant treatments. Group 3 (adjuvant FOLFOX) served as the control arm.

### 2.5. Statistical Analysis

**Figure 1:**
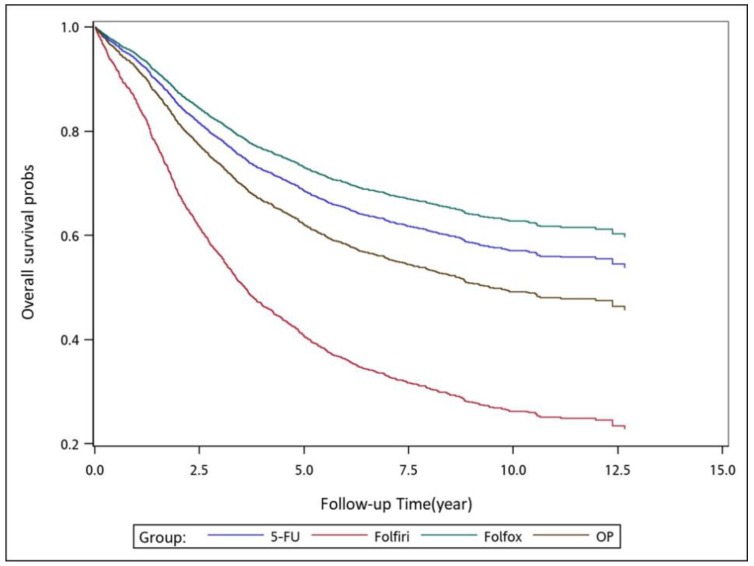
Predicted Cox proportional hazards curves for the overall survival of patients with high-risk stage II or III colon cancer who received different adjuvant chemotherapy regimens.

**Figure 2:**
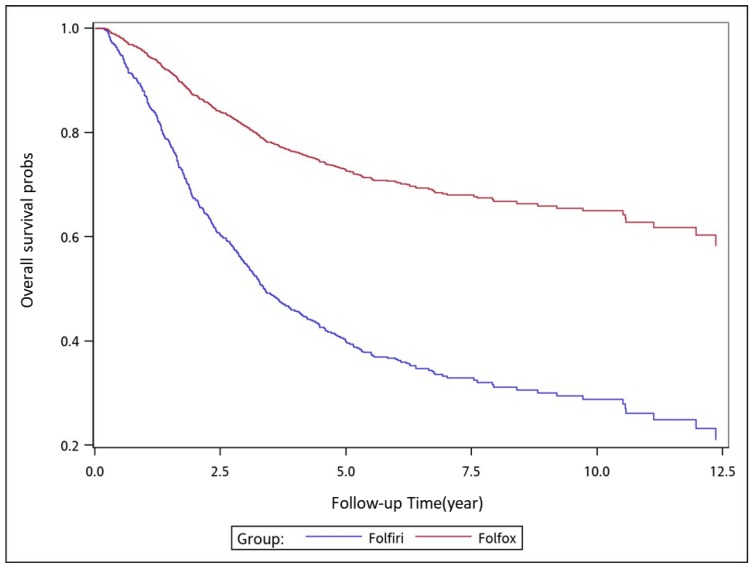
Predicted Cox proportional hazards curves for the overall survival of patients with high-risk stage II or III colon cancer who received adjuvant FOLFOX compared with those who received adjuvant FOLFIRI.

**Figure 3:**
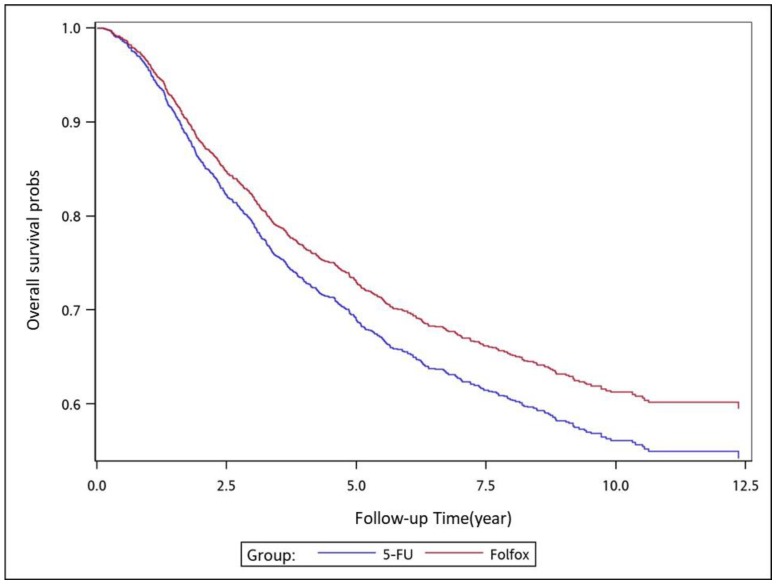
Predicted Cox proportional hazards curves for the overall survival of patients with high-risk stage II or III colon cancer who received adjuvant FOLFOX compared with those who received adjuvant fluoropyrimidine.

**Figure 4:**
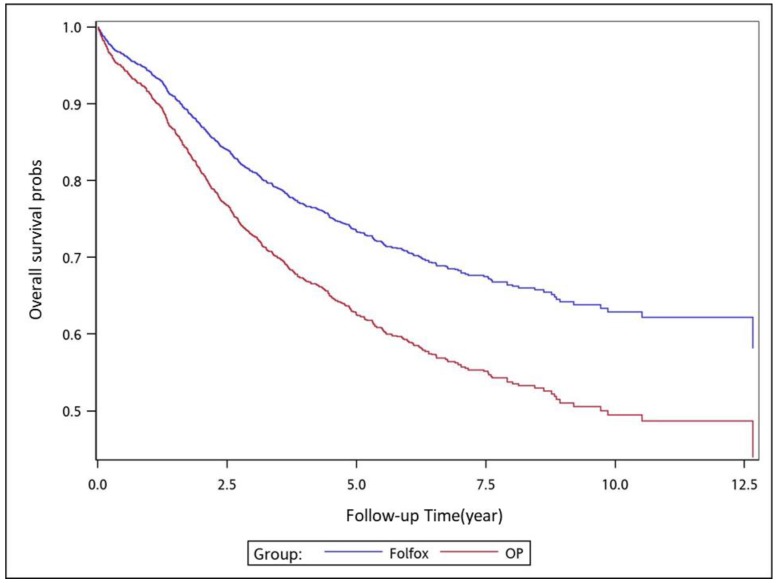
Predicted Cox proportional hazards curves for the overall survival of patients with high-risk stage II or III colon cancer who received adjuvant FOLFOX compared with those who received surgery alone.

**Figure 5:**
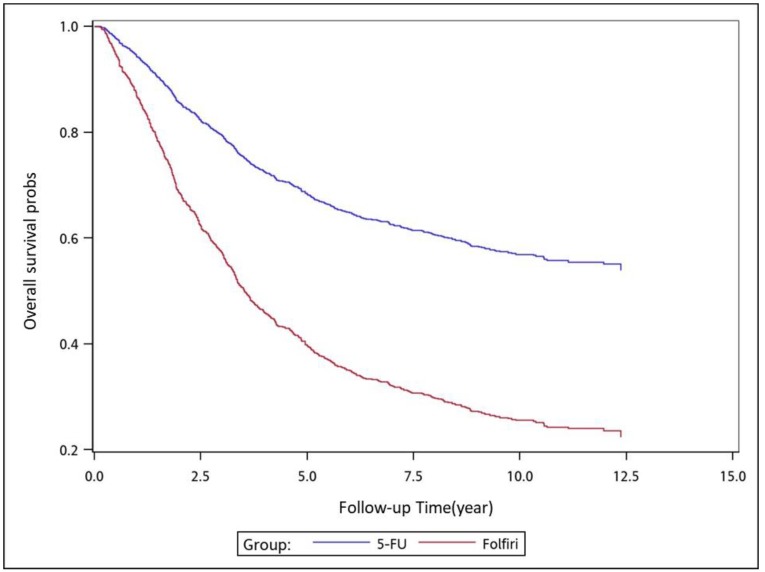
Predicted Cox proportional hazards curves for the overall survival of patients with high-risk stage II or III colon cancer who received adjuvant fluoropyrimidine compared with those who received adjuvant FOLFIRI.

**Figure 6:**
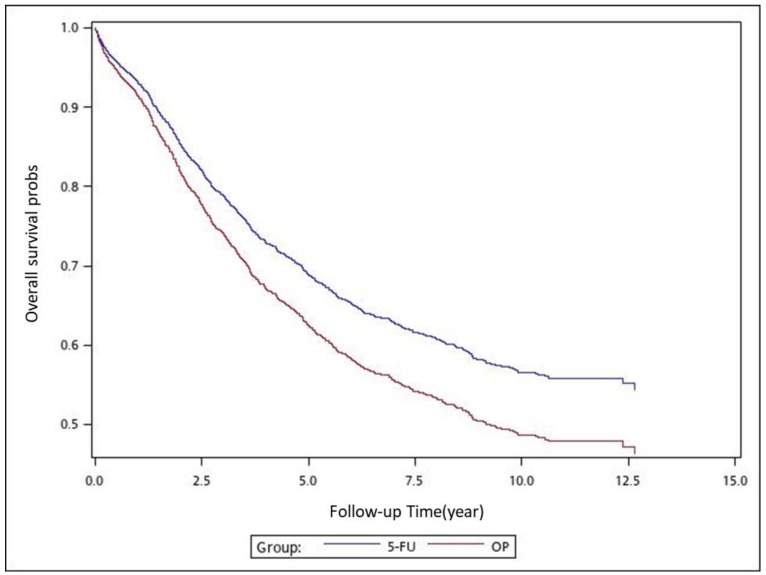
Predicted Cox proportional hazards curves for the overall survival of patients with high-risk stage II or III colon cancer who received adjuvant fluoropyrimidine compared with those who received surgery alone.

**Figure 7:**
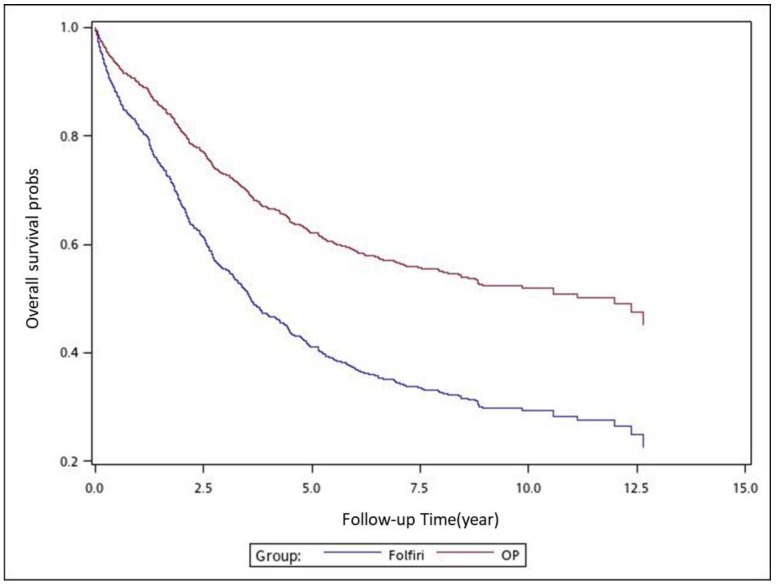
Predicted Cox proportional hazards curves for the overall survival of patients with high-risk stage II or III colon cancer who received adjuvant FOLFIRI compared with those who received surgery alone.

We used analysis of variance (ANOVA) to compare the mean among the 4 treatment groups, and Kruskal–Wallis test (K-W test) to compare the median among the 4 treatment groups. We used the Chi-square test to examine the relationships between treatment groups and categorical factors, such as sex, age group, stages, tumor locations, and CCI groups. We used the Cox proportional hazards model with a robust variance estimator to calculate the hazard ratios (HRs) to determine whether factors, such as adjuvant chemotherapy regimens, sex, age, pathologic stage, tumor location, and CCI score, were significant independent predictors (Table 1). We controlled for independent predictors in our analysis; mortality in the adjuvant chemotherapy groups served as the endpoint, with group 3 (adjuvant FOLFOX) serving as the control group. The cumulative incidence of death was estimated using the Cox proportional hazards model with a robust variance estimator of overall survival (OS) in patients who received the aforementioned adjuvant chemotherapy regimens; the model was also used in subgroup analyses with respect to pathologic stage, tumor location, sex, and age (Table 2, Table 3, Table 4 and Table 5). After adjusting for confounders, we also used the Cox proportional hazards model with a robust variance estimator to model the time between the index date and all-cause mortality in patients who received the aforementioned adjuvant chemotherapy regimens. In the multivariate analysis, we adjusted the HRs for sex, age, pathologic stage, tumor location, and CCI score. All analyses were performed using SAS software (version 9.3; SAS, Cary, NC, USA). We considered a two-tailed *p* value of <0.05 as indicating statistical significance. We used the Kaplan–Meier method to estimate the cumulative incidence of death, and we applied the log-rank test to determine differences among the adjuvant therapy regimens (Figure 1, Figure 2, Figure 3, Figure 4, Figure 5, Figure 6 and Figure 7).

## 3. Results

After applying the exclusion criteria and PSM algorithm, we included 3704 patients with high-risk stage II or III colon adenocarcinoma. Of these patients, 1140 were treated with surgery alone (group 1), 1144 were administered adjuvant fluoropyrimidine alone (group 2), 1134 received adjuvant FOLFOX (group 3), and 286 received adjuvant FOLFIRI (group 4). The mean ages of the patients in groups 1, 2, 3, and 4 were 59.9, 58.4, 58.7, and 58.4 years, respectively, and the median follow-up durations in these groups were 3.9, 7.1, 4.7, and 3.5 years, respectively. The 10-year age intervals as well as the CCI scores were nearly balanced for the four groups (Table 6). The number of deaths for the surgery-alone group is 388; for the adjuvant fluoropyrimidine-alone group, it is 436; for the adjuvant FOLFOX group, it is 278; and for the adjuvant FOLFIRI group, it is 172. The number of deaths overall is 1274. The AJCC pathologic stages were similar in the adjuvant treatment groups. Sex, tumor location, chemotherapy cycles, treatment duration, and CCI score were almost identical in the corresponding treatment groups after PSM (Table 6). Follow-up durations were not matched in the analysis because survival times were inconsistent in the treatment groups (Table 6).

According to the multivariate Cox regression analysis, adjuvant chemotherapy regimens were significant independent predictors of OS (Table 1). Both univariate and multivariate Cox regression analyses indicated that adjuvant FOLFOX was a significant independent prognostic factor for relatively high OS. Both analyses revealed that the adjusted HRs (aHRs, as well as the corresponding 95% confidence intervals (Cis)) for groups 1, 2, and 4 relative to group 3 were 1.55 (1.32 to 1.82), 1.22 (1.05 to 1.43), and 2.97 (2.43 to 3.63), respectively (Table 1). Moreover, in both analyses, the significant independent prognostic risk factors for poor OS were the male sex, age of ≥60 years, pathologic stage III, right-side colon cancer, and CCI score of ≥4 (Table 1).

We applied the Cox proportional hazards model with a robust variance estimator to evaluate the cumulative incidence of death in the patients in the various groups; we also performed subgroup analyses with respect to pathologic stage, tumor location, sex, and age. The results also indicated that regardless of age, sex, or tumor location, adjuvant FOLFOX was superior to adjuvant FOLFIRI, surgery alone, and adjuvant fluoropyrimidine alone (Table 3, Table 4 and Table 5). However, for high-risk stage II cancer having high-risk pathologic features, adjuvant fluoropyrimidine alone was superior to adjuvant FOLFOX (Table 2). Furthermore, multivariate Cox regression analyses revealed that the aHR (95% CI) for group 2 was 0.52 (0.30 to 0.89) relative to group 3 (Table 2), indicating that fluoropyrimidine alone was a significant independent prognostic risk factor for superior OS.

To analyze the risk of death associated with the various adjuvant chemotherapy regimens, we employed the predicted Cox proportional hazard curves for OS estimates for the patients (Figure 1, Figure 2, Figure 3, Figure 4, Figure 5, Figure 6 and Figure 7). To investigate the risk of death after receiving an adjuvant chemotherapy regimen, we compared the regimens (Figure 2, Figure 3, Figure 4, Figure 5, Figure 6 and Figure 7). Group 3 had the highest survival rate, followed by groups 2, 1, and 4. (Figure 1, Figure 2, Figure 3, Figure 4, Figure 5, Figure 6 and Figure 7). According to our data and sample size with a significance level of 0.05, the power of comparing the adjuvant fluoropyrimidine group and adjuvant FOLFOX group is 77%, the power of comparing the adjuvant fluoropyrimidine group and surgery-alone group is 94%, and the power of the other pair of comparisons is greater than 99%. If we consider the multiple testing and change the significance level to 0.0083, the power of comparing the adjuvant fluoropyrimidine group and adjuvant FOLFOX group is 52%, the power of comparing the adjuvant fluoropyrimidine group and surgery-alone group is 80%, and the power of the other pair of comparisons is still greater than 99%. Therefore, the power is available given the current sample size.

## 4. Discussion

For patients who have undergone potentially curative resection of advanced stage colon adenocarcinoma, the goal of adjuvant therapy is to eradicate micrometastases, thereby reducing the likelihood of disease recurrence and increasing the cure rate [5,6,8]. Adjuvant chemotherapy has been clearly demonstrated to have survival benefits in pathologic stage III (node-positive) disease, engendering an approximately 30% reduction in the risk of local recurrence and an approximately 20% to 30% increase in OS; however, its benefits in stage II disease remain controversial [5,6,7,29]. Data from randomized trials and meta-analyses have indicated that although fluoropyrimidine-based chemotherapy therapy benefits patients with resected stage II tumors, it does not engender a >5% absolute improvement in 5-year survival [30,31,32]. In the current study, we included all patients with high-risk stage II colon adenocarcinoma having high-risk pathologic features; thus, we could determine the optimal chemotherapy regimen. We suggest that adjuvant fluoropyrimidine-based chemotherapy alone is sufficient for high-risk stage II colon adenocarcinoma having high-risk pathologic features (Table 2).

According to our review of the relevant literature, our study is the first to evaluate the effects of FOLFOX, FOLFIRI, and adjuvant fluoropyrimidine-based chemotherapy alone for resected stage II or III colon cancer (Table 1). Only one US intergroup trial (E3201) compared adjuvant FOLFOX with FOLFIRI for the treatment of rectal cancer [33] (but not colon adenocarcinoma); however, the trial was terminated due to a competing trial (E5204). We discovered that adjuvant FOLFIRI is inferior to other adjuvant chemotherapy regimens, regardless of age, sex, pathologic stage, or tumor location (Table 2, Table 3, Table 4 and Table 5).

Oxaliplatin is the only platinum-type drug with activity in colorectal cancer; it is used only in combination with a fluoropyrimidine drug [7]. The survival benefit of adding oxaliplatin to adjuvant fluoropyrimidine-based therapy after the resection of node-positive stage III colon cancer tumors has been demonstrated in randomized trials that have typically enrolled younger, healthier, and less racially diverse patients; this benefit has also been supported by a combined analysis of data from five randomized trials [29] and by a large analysis of five observational cohorts of patients treated at the community level in diverse practice settings, including older and minority patients and patients with higher levels of comorbidity [34]. The benefit of adding oxaliplatin to fluoropyrimidine was first suggested in the MOSAIC trial, which randomly assigned 2246 patients with resected stage II (40%) or III colon cancer to a 6-month course of oxaliplatin-fluoropyrimidine treatment [5,7]. NSABP C-07 randomly assigned 2407 patients with stage II (29%) or III colon cancer to groups treated with fluoropyrimidines alone or with fluoropyrimidines plus oxaliplatin. The trial reported that the combined treatment with oxaliplatin was associated with more favorable outcomes compared with fluoropyrimidines alone [6]. NSABP C-07 indicated that adding oxaliplatin to fluoropyrimidines resulted in superior disease-free survival when compared with fluoropyrimidines alone, but the difference in OS was not statistically significant [6]. Our outcomes are comparable to those reported by the MOSAIC trial, which revealed that adjuvant FOLFOX was superior to adjuvant fluoropyrimidine alone in the treatment of resected stage III colon adenocarcinoma (Table 2). If patients with stage III colon adenocarcinoma cannot tolerate the toxicities of adjuvant FOLFOX, adjuvant fluoropyrimidine alone might be an alternative that is superior to surgery alone.

Risk stratifications for stage II colon cancer have yet to be established or commonly applied globally. Specifically, no clear risk stratifications exist for defining high-risk stage II colon cancer. In addition, immunohistochemistry, specific molecular tests, microsatellite instability testing, and genetic tests (such as mismatch repair enzyme status, *BRAF* mutation, and microsatellite instability-high) are not affordable for conducting routine examination in developing or other countries. Molecular features of tumors are generally used to guide decision making for adjuvant chemotherapy in patients with stage II disease, although evidence supporting this practice is still weak [35,36,37,38,39,40,41,42,43,44,45]. In most countries (including Taiwan), the most common, reliable, and affordable methods of determining risk features are examinations of high-risk clinicopathologic features and the tumor, node, and metastasis (TNM) stage [46,47,48,49,50,51,52].

In the current study, we selected high-risk stage II colon adenocarcinoma having high-risk pathologic features to estimate the effects of different adjuvant chemotherapy regimens. Notably, we observed that adjuvant FOLFOX did not have survival benefits relative to surgery alone (Table 2). Additionally, the survival benefits of adjuvant fluoropyrimidine alone were superior to those of adjuvant FOLFOX alone, adjuvant FOLFIRI, and surgery alone (Table 2). Accordingly, our study is the first to demonstrate that adjuvant fluoropyrimidine alone is sufficient and engenders superior survival rates relative to nonadjuvant chemotherapy, adjuvant FOLFOX, and adjuvant FOLFIRI in high-risk resected stage II colon adenocarcinoma with high-risk clinicopathologic features.

As presented in Table 1, we observed that significant independent prognostic risk factors for poor OS were the male sex, age of >60 years, CCI scores of ≥4, and right-side colon adenocarcinoma [53]. These poor prognostic factors are consistent with those outlined in previous studies [9,10,11,12,13,14,54,55]. Therefore, we conducted subgroup analyses with respect to sex, tumor location, and pathologic stage (Table 2, Table 3, Table 4 and Table 5). The trends of survival rates under different adjuvant chemotherapy regimens remained unchanged (with similar results to those in Table 1). Adjuvant FOLFOX was superior to adjuvant FOLFIRI, adjuvant fluoropyrimidine alone, and surgery alone, regardless of age, sex, or tumor location. The four regimens can be ordered (in descending order) as follows in terms of their associated survival rates in patients with resected stage III colon adenocarcinoma, regardless of age, tumor location, or sex: Adjuvant FOLFOX, adjuvant fluoropyrimidine alone, surgery alone, and adjuvant FOLFIRI (Table 3, Table 4 and Table 5). The trends of survival rates associated with the adjuvant chemotherapy regimens differed only for high-risk stage II cancer with high-risk clinicopathologic features. Adjuvant fluoropyrimidine alone yielded the best results for high-risk stage II resected colon adenocarcinoma (Table 2).

The essential principles of treating colon cancer are the same for both younger and older patients. However, older patients cannot tolerate adjuvant chemotherapy because they may have age-related organ function decline and comorbid conditions that potentially limit life expectancy [9,10,11,12]. We applied the age-stratified Cox proportional hazard regression model with a robust variance estimator to determine the risk of death among patients with colon adenocarcinoma who received the aforementioned adjuvant therapeutic regimens. As indicated in Table 3, our results showed that adjuvant FOLFOX was superior to the other chemotherapy regimens and non-adjuvant chemotherapy. Older patients derived as much benefit from adjuvant fluoropyrimidine alone as did younger patients; older patients may even derive incremental benefits from the addition of oxaliplatin (Table 3). Although pooled analyses have indicated a modest increase in the rate of severe hematologic toxicity in healthy older adults, other toxicities are not necessarily worse when close attention is paid to regimen selection [56,57]. According to our findings, we recommend routine adjuvant chemotherapy, particularly adjuvant FOLFOX, for healthy older patients with stage III or high-risk stage II colon cancer (Table 3). This is the first study to establish that adjuvant chemotherapy is the most optimal regimen for elderly patients with resected high-risk stage II or stage III colon adenocarcinoma, followed by adjuvant FOLFOX and adjuvant fluoropyrimidine alone.

Adjuvant irinotecan-containing regimens cannot be considered a standard approach for patients requiring adjuvant chemotherapy for colon adenocarcinoma [58,59,60]. In previous studies, adjuvant irinotecan-containing chemotherapy has been studied in three separate trials, all of which demonstrate no benefit for either bolus or infusional irinotecan-containing chemotherapy and increase both lethal and nonlethal toxicity compared with fluoropyrimidine alone [58,59,60]. Irinotecan-containing regimens are considered for metastatic colorectal cancer instead of adjuvant chemotherapy regimens [61,62,63]. Furthermore, rates of grade 3 or 4 toxicity were also significantly higher with irinotecan, with higher rates of neutropenia, febrile neutropenia, and diarrhea [64]. Regimens combining irinotecan with bolus fluoropyrimidine and leucovorin are even more toxic and twice as likely to have severe neutropenia [64,65]. Irinotecan-containing regimens were not compared on rates of infection or hospitalization by age, or what proportion of the patients with fatal chemotherapy toxicity were older adults [64,65]. Therefore, patients in the adjuvant FOLFIRI group have a lower overall survival than the other groups and even compared to the surgery-alone group (Table 1, Table 2, Table 3, Table 4 and Table 5), because adjuvant FOLFIRI would be too toxic for relatively better survival rates in stage II-III colon cancer than metastatic colon cancer patients.

The strength of this study is that it is the first large-cohort study to apply PSM to estimate the OS benefits of adjuvant chemotherapy regimens for patients with high-risk stage II colon adenocarcinoma having high-risk pathologic features. Furthermore, our findings demonstrate that adjuvant FOLFOX yields the most favorable OS in elderly patients (≥60 years; the median age of patients with colon cancer in Taiwan is 60 years) with high-risk stage II or stage III colon adenocarcinoma when compared with adjuvant FOLFIRI, adjuvant fluoropyrimidine alone, and surgery alone. We suggest adjuvant FOLFOX in patients (of both sexes) with stage III colon adenocarcinoma (regardless of tumor location). The pathologies, covariates, and pathologic stages in our study were more homogenous than those in other studies. The outcomes in our study could serve as a useful reference for clinical practice and future clinical trials.

This study has some limitations. First, we could not determine the toxicity induced by the various adjuvant regimens. Therefore, our treatment-related mortality estimates may have been biased. However, a previous study demonstrated that adjuvant FOLFOX was associated with more complications and higher toxicity compared with surgery alone and adjuvant fluoropyrimidine alone [5,7]. Thus, in our study, the survival benefits of adjuvant FOLFOX could only be underestimated. Second, because all patients with colon adenocarcinoma were enrolled from an Asian population, the corresponding ethnic susceptibility remains unclear. Accordingly, our results should be cautiously extrapolated to non-Asian populations. Third, in our study, we did not have molecular data for patients with colon adenocarcinoma. However, despite encouraging preliminary data linking molecular findings to prognosis and potentially better prognostic stratifications relative to the TNM stage alone, no single molecular marker, multiple marker profile, or gene expression panel of predictive utility has emerged [35,36,37,38,39,40,41,42,43,44,45]. Fourth, the diagnoses of all comorbid conditions were based on ICD-9-CM codes. Nevertheless, the Taiwan Cancer Registry Administration randomly reviews charts and interviews patients to verify the accuracy of the diagnoses, and hospitals with outlier chargers or practices may be audited and subsequently penalized heavily if malpractice or discrepancies are identified. Therefore, to obtain crucial information on population specificity and disease occurrence, a large-scale randomized trial comparing carefully selected patients undergoing suitable treatment is essential. Finally, the Cancer Registry database does not contain information regarding dietary habits, socioeconomic status, or body mass index, all of which may be risk factors for mortality. Nevertheless, considering the magnitude and statistical significance of the observed effects in this study, these limitations are unlikely to affect the conclusions.

## 5. Conclusions

Adjuvant fluoropyrimidine alone was the most optimal regimen for patients with high-risk stage II colon adenocarcinoma having high-risk pathologic features compared with the other adjuvant chemotherapy regimens. Adjuvant FOLFOX could be the optimal regimen for patients with pathologic stage III colon adenocarcinoma, regardless of age, sex, or tumor location.

## Figures and Tables

**Table 1:** Cox proportional hazard regression model with a robust variance estimator for evaluating the risk of death among patients with colon adenocarcinoma who received different adjuvant therapeutic regimens.

Univariate Analysis				Multivariate Analysis *			
Variables	HR	95% CI	*p*-Value		aHR	95% CI	*p*-Value
**Adjuvant Treatments**							
FOLFOX	1			FOLFOX	1		
Surgery alone	1.58	(1.35–1.86)	<0.0001	Surgery alone	1.55	(1.32–1.82)	<0.0001
Fluoropyrimidine	1.27	(1.08–1.48)	0.0032	Fluoropyrimidine	1.22	(1.05–1.43)	0.012
FOLFIRI	3.06	(2.5–3.76)	<0.0001	FOLFIRI	2.97	(2.43–3.63)	<0.0001
**Age**							
<40	1			<40	1		
40–49	0.95	(0.82–1.1)	0.4923	40–49	1.02	(0.78–1.32)	0.9021
50–59	1	(0.87–1.15)	0.9963	50–59	1.03	(0.81–1.3)	0.8303
60–69	1.23	(1.08–1.4)	0.002	60–69	1.29	(1.04–1.6)	0.0194
70–79	2.03	(1.79–2.31)	<0.0001	70–79	2.07	(1.66–2.58)	<0.0001
≥80	3.63	(3.19–4.14)	<0.0001	≥80	3.01	(2.25–4.02)	<0.0001
**Sex**							
Female	1			Female	1		
Male	1.23	(1.18–1.27)	<0.0001	Male	1.19	(1.06–1.32)	0.0022
**AJCC Pathologic stages**							
High risk IIB–IIC	1			2	1		
III	1.56	(1.49–1.63)	<0.0001	3	1.52	(1.25–1.84)	<0.0001
**Tumor Locations**							
Left	1			Left	1		
Transverse	1.61	(1.45–1.79)	<0.0001	Transverse	1.7	(1.42–2.03)	<0.0001
Right	1.16	(1.11–1.21)	<0.0001	Right	1.2	(1.07–1.35)	0.0014
**CCI**				CCI			
0	1			0	1		
1	1.13	(1.02–1.24)	0.0179	1	1.08	(0.82–1.42)	0.5911
2	1.24	(1.12–1.37)	<0.0001	2	1.07	(0.77–1.49)	0.6951
3	1.43	(1.29–1.58)	<0.0001	3	1.21	(0.91–1.61)	0.1855
≥4	1.94	(1.78–2.11)	<0.0001	≥4	1.49	(1.2–1.85)	0.0003

* All aforementioned variables were used in multivariate analysis. CCI, Charlson comorbidity index; CI, confidence interval; aHR, adjusted hazard ratio; AJCC, American Joint Committee on Cancer; FOLFOX, Folinic acid, Fluorouracil, Oxaliplatin; FOLFIRI, Folinic acid, Fluorouracil, Irinotecan.

**Table 2:** AJCC stage-stratified Cox proportional hazard regression model with a robust variance estimator for evaluating the risk of death among patients with colon adenocarcinoma with different pathologic stages who received various adjuvant therapeutic regimens.

High-Risk Stage IIB-IIC				Stage III			
Adjuvant Treatments	aHR *	95% CI	*p*-Value	Adjuvant Treatments	aHR *	95% CI	*p*-Value
FOLFOX	1			FOLFOX	1		
Surgery alone	0.87	(0.52–1.43)	0.5709	Surgery alone	1.67	(1.40–1.99)	<0.0001
Fluoropyrimidine	0.52	(0.30–0.89)	0.0179	Fluoropyrimidine	1.38	(1.16–1.63)	<0.0001
FOLFIRI	1.61	(0.85–3.04)	0.1470	FOLFIRI	3.27	(2.64–4.07)	<0.0001

* All the aforementioned variables in Table 1 were used in multivariate analysis. CCI, Charlson comorbidity index; CI, confidence interval; aHR, adjusted hazard ratio; AJCC, American Joint Committee on Cancer; FOLFOX (Folinic acid, Fluorouracil, Oxaliplatin); FOLFIRI (Folinic acid, Fluorouracil, Irinotecan).

**Table 3:** Age-stratified Cox proportional hazard regression model with a robust variance estimator for evaluating the risk of death among patients with colon adenocarcinoma who received various adjuvant therapeutic regimens.

≤60 Years Old				>60 Years Old			
Adjuvant Treatments	aHR *	95% CI	*p*-Value	Adjuvant Treatments	aHR *	95% CI	*p*-Value
FOLFOX	1			FOLFOX	1		
Surgery alone	1.58	(1.22–2.05)	0.0006	Surgery alone	1.63	(1.32–2.02)	<0.0001
Fluoropyrimidine	1.31	(1.02–1.67)	0.0335	Fluoropyrimidine	1.27	(1.03–1.57)	0.0263
FOLFIRI	3.66	(2.7–4.97)	<0.0001	FOLFIRI	2.75	(2.07–3.65)	<0.0001

* All the aforementioned variables in Table 1 were used in multivariate analysis. CCI, Charlson comorbidity index; CI, confidence interval; aHR, adjusted hazard ratio; AJCC, American Joint Committee on Cancer; FOLFOX (Folinic acid, Fluorouracil, Oxaliplatin); FOLFIRI (Folinic acid, Fluorouracil, Irinotecan).

**Table 4:** Sex-stratified Cox proportional hazard regression model with a robust variance estimator for evaluating the risk of death among patients with colon adenocarcinoma who received various adjuvant therapeutic regimens.

Male				Female			
Adjuvant Treatments	aHR *	95%CI	*p*-Value	Adjuvant Treatments	aHR *	95%CI	*p*-Value
FOLFOX	1			FOLFOX	1		
Surgery alone	1.7	(1.36–2.11)	<0.0001	Surgery alone	1.47	(1.15–1.87)	0.0019
Fluoropyrimidine	1.48	(1.2–1.83)	0.0003	Fluoropyrimidine	1.04	(1.02–1.32)	0.0492
FOLFIRI	3.18	(2.41–4.19)	<0.0001	FOLFIRI	3.02	(2.23–4.09)	<0.0001

* All the aforementioned variables in Table 1 were used in multivariate analysis. CCI, Charlson comorbidity index; CI, confidence interval; aHR, adjusted hazard ratio; AJCC, American Joint Committee on Cancer; FOLFOX (Folinic acid, Fluorouracil, Oxaliplatin); FOLFIRI (Folinic acid, Fluorouracil, Irinotecan).

**Table 5:** Tumor location-stratified Cox proportional hazard regression model with a robust variance estimator for evaluating the risk of death among patients with colon adenocarcinoma who received various adjuvant therapeutic regimens.

Left				Transverse				Right			
Adjuvant Treatments	aHR *	95%CI	*p*-Value	Adjuvant Treatments	aHR *	95%CI	*p*-Value	Adjuvant Treatments	aHR *	95%CI	*p*-Value
FOLFOX	1			FOLFOX	1			FOLFOX	1		
Surgery alone	1.23	(1.16–1.95)	0.0023	Surgery alone	1.32	(1.26–4.35)	0.0009	Surgery alone	1.47	(1.18–1.84)	0.0007
Fluoropyrimidine	1.17	(1.07–1.66)	0.0076	Fluoropyrimidine	1.12	(1.08–2.54)	0.0203	Fluoropyrimidine	1.18	(1.06–1.45)	0.0101
FOLFIRI	3.32	(2.38–4.65)	<0.0001	FOLFIRI	3.58	(1.37–9.36)	0.0093	FOLFIRI	2.7	(2.08–3.5)	<0.0001

* All the aforementioned variables in Table 1 were used in multivariate analysis. CCI, Charlson comorbidity index; CI, confidence interval; aHR, adjusted hazard ratio; AJCC, American Joint Committee on Cancer; FOLFOX (Folinic acid, Fluorouracil, Oxaliplatin); FOLFIRI (Folinic acid, Fluorouracil, Irinotecan).

**Table 6:** Characteristics of patients with colon adenocarcinoma who received surgery along with different adjuvant therapeutic regimens and their propensity score-matched cohort.

Variables	Surgery Alone	Adjuvant Fluoropyrimidine Alone	Adjuvant FOLFOX	Adjuvant FOLFIRI	*p*-Value
	N = 1140 (%)	N = 1144 (%)	N = 1134 (%)	N = 286 (%)	
**Sex**					0.9988 ^+^
Male	603 (52.9)	604 (52.8)	596 (52.6)	151 (52.8)	
Female	537 (47.1)	540 (47.2)	538 (47.4)	135 (47.2)	
**Age, mean (SD)**	59.9 (12.5)	58.4 (13.3)	58.7 (12.2)	58.4 (13)	0.0273 ^#^
					0.7508 ^+^
<40	68 (6)	107 (9.4)	90 (7.9)	24 (8.4)	
40–49	178 (15.6)	184 (16.1)	184 (16.2)	46 (16.1)	
50–59	290 (25.4)	266 (23.3)	281 (24.8)	68 (23.8)	
60–69	363 (31.8)	359 (31.4)	359 (31.7)	91 (31.8)	
70–79	209 (18.3)	196 (17.1)	189 (16.7)	49 (17.1)	
≥80	32 (2.8)	32 (2.8)	31 (2.7)	8 (2.8)	
**AJCC Pathologic stages**					0.9679 ^+^
High-risk IIB-IIC	152 (13.3)	152 (13.3)	144 (12.7)	38 (13.3)	
III	988 (86.7)	992 (86.7)	990 (87.3)	248 (86.7)	
**Tumor locations**					0.5438 ^+^
Left	525 (46.1)	509 (44.5)	504 (44.4)	127 (44.4)	
Transverse	61 (5.4)	45 (3.9)	46 (4.1)	14 (4.9)	
Right	554 (48.6)	590 (51.6)	584 (51.5)	145 (50.7)	
CCI					0.9994 ^+^
0	84 (9.8)	94 (11)	93 (10.9)	25 (11.7)	
1	111 (12.9)	111 (13)	105 (12.3)	26 (12.2)	
2	59 (6.9)	63 (7.4)	55 (6.4)	16 (7.5)	
3	25 (2.9)	24 (2.8)	27 (3.2)	6 (2.8)	
≥4	861 (75.5)	852 (74.5)	854 (75.3)	213 (74.5)	
**CCI**					
Mean (SD)	5.9 (3.3)	5.7 (3.2)	5.9 (3.2)	6 (3.3)	0.3447 ^#^
Median (IQR)	6 (4)	6 (5)	7 (4)	7 (5)	0.1167 *
Treatment duration (days), median (IQR)	−	240 (55)	224 (53)	245 (52)	0. 2346 *
Total cycles of Chemotherapy					
Median (IQR)	−	12 (3)	12 (2)	12 (2)	0.8883 *
Follow-up time (years)					
Mean (SD)	5 (3.2)	7.4 (4)	5.1 (2.4)	4.8 (3.4)	<0.0001 ^#^
Median (IQR)	3.9 (4.7)	7.1 (7.3)	4.7 (3.6)	3.5 (3.7)	<0.0001 *

Obs, observation; CCI, Charlson comorbidity index; SD, standard deviation; IQR, interquartile range; AJCC, American Joint Committee on Cancer; FOLFOX, Folinic acid, Fluorouracil, Oxaliplatin; FOLFIRI, Folinic acid, Fluorouracil, Irinotecan. ^#^ ANOVA: Compare the mean among the four treatment groups; * Kruskal–Wallis test: Compare the median among the four treatment groups; ^+^ Chi-square test: Examine the relationships between treatment groups and categorical factors, such as sex, age group, stages, tumor locations, and CCI groups.

## Abbreviations

ICD-9-CM	International Classification of Diseases: Ninth Revision, Clinical Modification
AJCC	American Joint Committee on Cancer; HR, hazard ratio
CI	confidence interval
CCI	Charlson comorbidity index
OS	overall survival
PSM	propensity score–matched
FOLFOX	oxaliplatin, fluoropyrimidine, and leucovorin
FOLFIRI	folinic acid, fluorouracil, irinotecan
aHRs	adjusted HRs
MOSAIC	multicenter international study of oxaliplatin, fluoropyrimidine, and leucovorin in the adjuvant treatment of colon cancer
NSABP	national surgical adjuvant breast and bowel project
NCCN	National Comprehensive Cancer Network
TCRD	Taiwan Cancer Registry database
AJCC	American Joint Committee on Cancer
CCI	Charlson comorbidity index; PS, propensity score
TNM	tumor, node, metastasis
